# Human Serine Racemase: Key Residues/Active Site Motifs and Their Relation to Enzyme Function

**DOI:** 10.3389/fmolb.2019.00008

**Published:** 2019-03-13

**Authors:** Danielle L. Graham, Matthew L. Beio, David L. Nelson, David B. Berkowitz

**Affiliations:** Department of Chemistry, University of Nebraska-Lincoln, Lincoln, NE, United States

**Keywords:** D-serine, pyridoxal phosphate (PLP), serine racemase, racemization, elimination, mechanism, ATP, allosteric activation/regulation

## Abstract

Serine racemase (SR) is the first racemase enzyme to be identified in human biology and converts L-serine to D-serine, an important neuronal signaling molecule that serves as a co-agonist of the NMDA (N-methyl-D-aspartate) receptor. This overview describes key molecular features of the enzyme, focusing on the side chains and binding motifs that control PLP (pyridoxal phosphate) cofactor binding as well as activity modulation through the binding of both divalent cations and ATP, the latter showing allosteric modulation. Discussed are catalytically important residues in the active site including K56 and S84—the si- and re-face bases, respectively,—and R135, a residue that appears to play a critical role in the binding of both negatively charged alternative substrates and inhibitors. The interesting bifurcated mechanism followed by this enzyme whereby substrate L-serine can be channeled either into D-serine (racemization pathway) or into pyruvate (β-elimination pathway) is discussed extensively, as are studies that focus on a key loop region (the so-called “triple serine loop”), the modification of which can be used to invert the normal *in vitro* preference of this enzyme for the latter pathway over the former. The possible cross-talk between the PLP enzymes hSR and hCBS (human cystathionine β-synthase) is discussed, as the former produces D-serine and the latter produces H_2_S, both of which stimulate the NMDAR and both of which have been implicated in neuronal infarction pursuant to ischemic stroke. Efforts to gain a more complete mechanistic understanding of these PLP enzymes are expected to provide valuable insights for the development of specific small molecule modulators of these enzymes as tools to study their roles in neuronal signaling and in modulation of NMDAR function.

## Introduction

Pyridoxal phosphate (PLP) enzymes have been extensively studied owing to interest in both the molecular details of their chemistry and the physiologic importance of the reactions that they catalyze, particularly in the area of neuroactive amine homeostasis (Walsh, 1979; Toney, 2005). This review will focus on key residues, binding sites and catalytically important motifs of human serine racemase, and its mammalian counterparts. In the literature to date, there are several reviews on topics such as the importance of D-serine and serine racemase in glial neurotransmission (Mothet, 2008; Wolosker, 2011, 2018; Wolosker and Mori, 2012; Wolosker et al., 2016) and in neurodegeneration (Campanini et al., 2013; Coyle and Balu, 2018). In addition, recent reviews by Mozzarelli (Raboni et al., 2019) and by Mori (Mori, 2014) discuss the SR energy landscape and mechanism in the context of structure. The current review also builds upon previous reviews that discuss inhibition of hSR (Jirásková-Vanícková et al., 2011), in focusing upon key residues and structural motifs to consider in generating future inhibitors.

**Figure 1:**
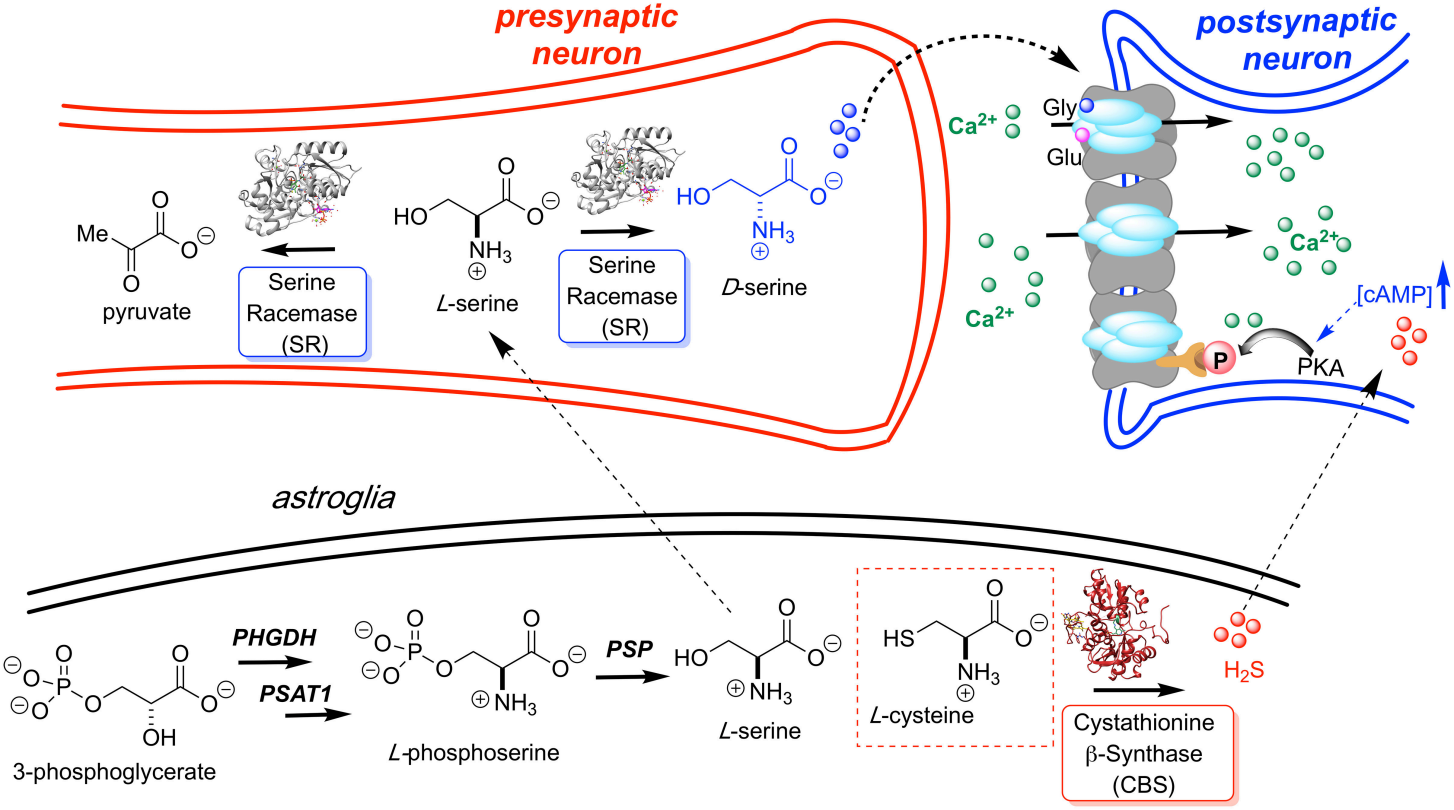
L-Serine is first produced in the astroglia from 3-phosphoglycerate by way of a three enzyme cascade involving phosphoglycerate dehydrogenase (PHGDH), phosphoserine aminotransferase 1 (PSAT1), and phosphoserine phosphatase (PSP). L-Serine is then transported to the presynaptic neuron where it is converted to D-Ser by hSR. D-Serine acts as a potent NMDAR co-agonist at the glycine site. H_2_S, generated by CBS, is also thought to activate the NMDA receptor. Models for H_2_S-NMDAR activation include a cAMP-dependent PKA-mediated phosphorylation and H_2_S- or sulfane sulfur-mediated NMDAR disulfide bond reduction (Kimura, 2000; Kimura et al., 2013).

Traditionally thought to be restricted almost exclusively to the domain of bacterial cell wall biosynthesis, D-amino acids are now clearly seen as playing important and as yet incompletely understood roles in human biology, particularly in neuronal signaling (Wolosker et al., 2008; Li et al., 2017; Weatherly et al., 2017; Du et al., 2018). Although the receptor is named after its ability to bind N-methyl-D-aspartate (NMDA), L-glutamate is the primary agonist of the NMDA receptor with D-serine (D-Ser), serving as co-agonist, as illustrated in Figure 1. The NMDAR operates primarily as a ligand-gated channel that dislodges a Mg^2+^ or Zn^2+^ ion, allowing for depolarization and Ca^2+^ influx. Ca^2+^ ions are critical for synaptic plasticity and appropriate levels of each agonist are required for neuronal homeostasis and long-term potentiation (LTP) associated with learning and memory.

Whereas D-serine binds to the so-called “glycine site” of the NMDAR, it displays >2 orders of magnitude more potent activation of the NMDAR than glycine (Gly) itself (Berger et al., 1998; Wolosker, 2007). Perhaps the best experiments demonstrating this are elegant *in vitro* measurements of miniature excitatory postsynaptic currents (mEPSCs). In response to coagonist stimulation, 0.3 μM D-serine produces a higher level of NMDA charge transfer than 30 μM glycine (Berger et al., 1998). Consistent with these observations, the crystal structures of the NR1 subunit of the NMDAR with bound D-Ser (PDB code: 1PB8) and with bound Gly (PDB code: 1PB7) demonstrate that the former ligand engages in several additional hydrogen bonds as compared with the latter (Furukawa and Gouaux, 2003). This topic has been more extensively reviewed elsewhere (Schell, 2004). Recent reports also show that D-Ser, and not Gly, is responsible for LTP in the visual cortex (Meunier et al., 2016), and demonstrate that D-Ser concentrations in compartments of the cerebellum are much more tightly controlled than those of Gly, with the former being concentrated in the neocortex where complex thinking is taking place (Suzuki et al., 2017).

At the turn of the millennium, it was established that biosynthesis of D-Ser is mediated by a PLP-dependent serine racemase enzyme. This constituted the first known example of a mammalian racemase enzyme (Wolosker et al., 1999; De Miranda et al., 2000). Interestingly, human serine racemase (hSR) has an apparent dual role as it funnels neuronal L-serine into bifurcating pathways toward either D-Ser (racemization) or pyruvate (β-elimination).

## Mechanism

**Figure 2:**
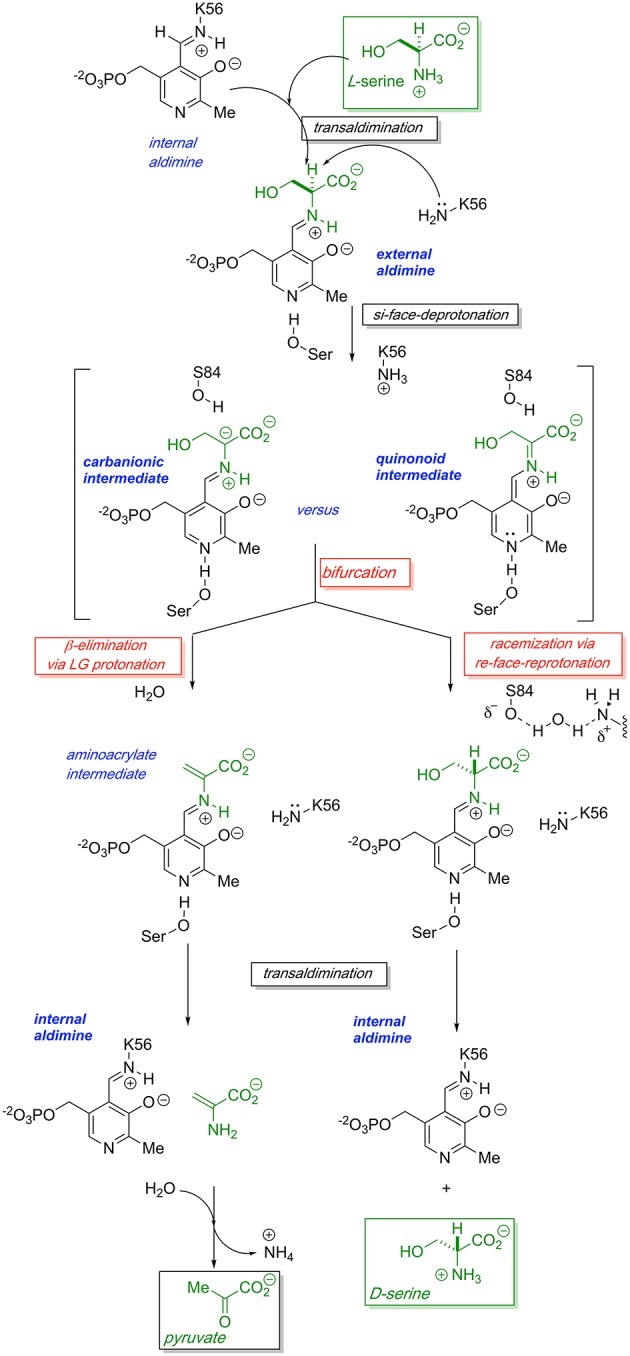
Proposed bifurcating mechanism of hSR showing the racemization reaction vs. the competing β-elimination reaction via a common carbanionic or quinonoid intermediate.

The generally accepted mechanism by which human SR catalyzes both the racemization of L-Ser to D-Ser and the elimination of L-Ser to pyruvate is illustrated schematically in Figure 2. Substrate L-Ser displaces K56 via an initial transaldimination reaction to form the external aldimine. The displaced K56 residue serves as the *si*-face base, α-deprotonating to yield a common carbanionic or quinonoid intermediate (see Discussion below). This is the point at which the path bifurcates with *re*-face reprotonation by S84 giving the racemization product, D-Ser, or with expulsion of the β-OH-leaving group, presumably following protonation, giving rise to an enamine that eventually is released as pyruvate, the β-elimination product. The wt-hSR enzyme displays an ~4-fold preference for the β-elimination pathway over racemization under *in vitro* steady-state enzyme kinetic conditions (Nelson et al., 2017). However, Toney and co-workers showed that this ratio can be significantly altered by selected mutations (Foltyn et al., 2005) as will be discussed. Moreover, given the number of important protein-protein interactions (PPI) that have been implicated for hSR *in vivo* (Fujii et al., 2006; Baumgart et al., 2007; Hikida et al., 2008; Ma et al., 2013, 2014), one must consider that these may influence hSR activity and the racemization to β-elimination ratio seen *in vivo* as well.

## Sequence Overview

**Figure 3:**
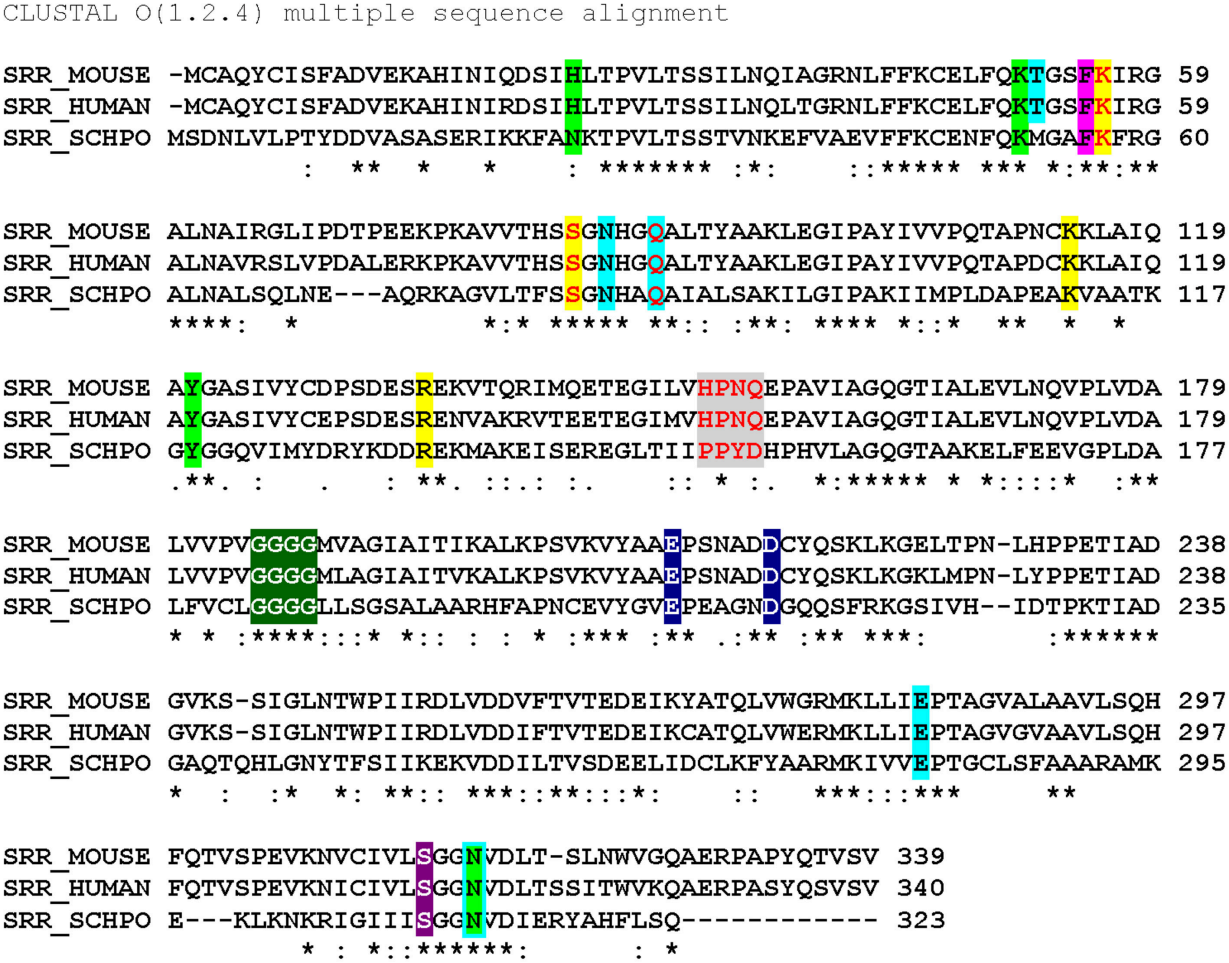
Sequence alignment (Clustal O) of SR from mouse, human and *S. pombe*. Color code: ATP binding site (lime); ATP hydrogen bonding network (cyan highlight or outline); π-stacking residue (magenta) and catalytic residues (yellow); “triple serine loop” region by analogy with aspartate racemase (gray); tetraglycine loop (green); divalent cation binding site (navy blue); pyridine-N-protonating residue (violet). Red letters indicate positions that have been mutated. *conserved residue: strongly similar residues. Weakly similar residues.

A global overview of SR primary structure with an eye toward highlighting key functional domains is presented in Figure 3. This review will discuss conserved motifs displayed there, including all the elements of the PLP binding pocket—the essential lysine residue, the tetraglycine loop for phosphate binding (Smith et al., 2010), the H-bond donor for the PLP ring nitrogen and the edge-to-face π-π interaction that serves to anchor the pyridine ring (Wang and Barger, 2012). The enzyme is stimulated by both divalent metal cation binding and ATP binding, each with established contact residues, with good evidence for allostery in the case of the nucleotide binding site. Finally, an interesting “triple Ser loop” is present that appears to have significant influence on the bifurcation, i.e., L-serine racemization vs. β-elimination activity.

## Divalent Metal Binding Site

**Figure 4:**
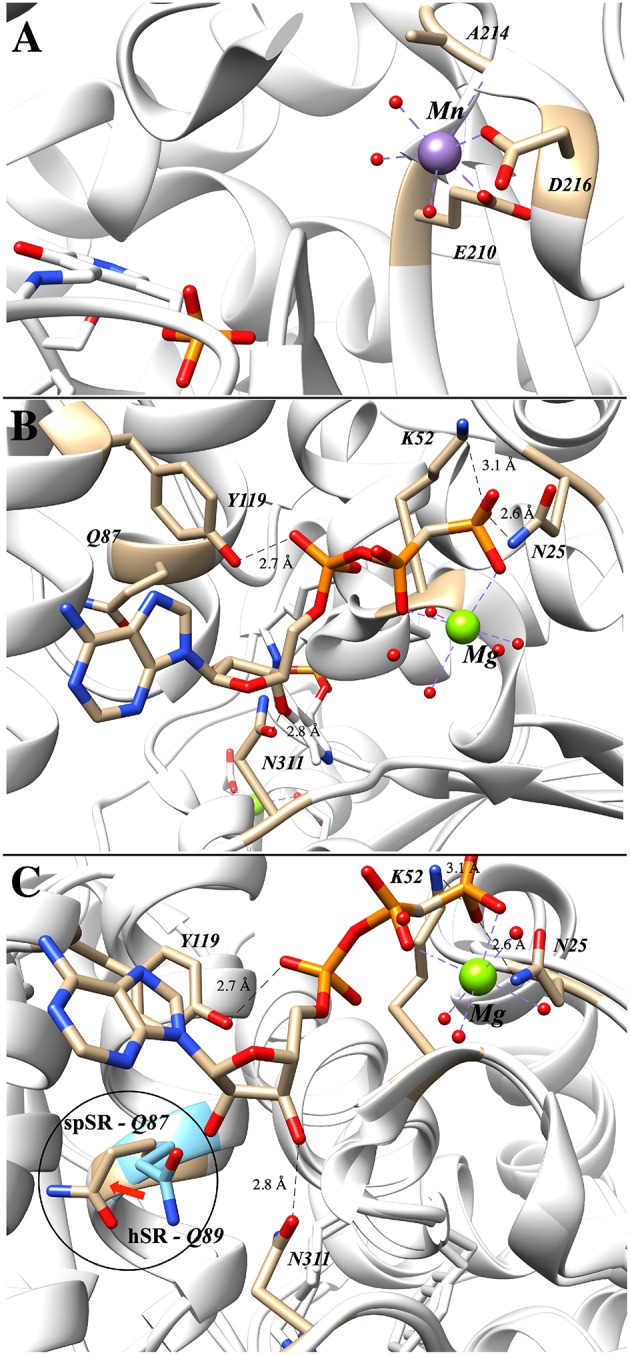
**(A)** Mn^2+^ occupying the metal binding site in hSR; both E210 and D216 are within 2.1 Å of the divalent metal (3L6B). **(B)** Binding of the second ATP–associated metal from the AMP-PCP-bound structure of *S. pombe* SR (1WTC). **(C)** Overlay of human (blue) and *S. pombe* (tan) SR highlighting the movement of Q89 upon ATP-binding (Q89 in hSR and Q87 in spSR).

The importance of divalent metal cation binding to hSR was first reported, in parallel, by the laboratories of Wolosker (De Miranda et al., 2002) and of Cook (Cook et al., 2002). The latter group performed the most extensive study of divalent cations, finding that Mn^2+^ leads to the highest increase in activity of the enzyme (153% @10 μM), followed by Ca^2+^ (134% @1 mM) and Mg^2+^ (112% @10 μM) relative to the purified hSR without divalent cation supplementation. The effect of divalent metal identity upon activity does not appear to be due to major structural changes, as evidenced by circular dichroism studies. Smith et al. have deposited the coordinates of several mammalian SR crystal structures including PDB code: 3L6B displayed in Figure 4A, a structure that highlights the formally octahedral divalent metal (Mn^2+^)-coordination sphere involving residues E210 and D216 (~2.1 Å metal-ligand bond lengths), an amide carbonyl and three water molecules. A similar divalent metal coordination environment is seen in the *S. pombe* SR enzyme (PDB code: 1WTC).

## ATP Binding Site

Of the crystal structures currently available for SR, the ATP-binding site is best seen in the *Schizosaccharomyces pombe* serine racemase (SpSR) structure (1WTC) that features a bound AMP-PCP ligand, a hydrolytically stable β,γ-CH_2_-phosphonate analog of ATP (Figure 4B). In this structure, residues K52 and N25 coordinate to the terminal phosphonate group and a Mg^2+^ ion bridges across the β,γ-phosphono groups. Tyrosine-119 appears to be engaged in hydrogen bonding interactions with the proximal phosphate, and Asn311 appears to be engaged in a similar H-bond with the 3'-hydroxyl group of the ribose.

It is well-known that ATP-binding leads to enhanced catalytic activity for SR; an overlay of the ATP-free structure (e.g., 3L6B) with the ATP bound structure (1WTC) provides evidence that this amounts to allosteric activation (Figure 4C). Specifically, it has been argued that allostery arises through an extensive hydrogen binding network (T52, N86, Q89, E283, N316) connecting the ATP ribose 3'-hydroxyl group to the active site (T52 corresponds to M53 in SpSR). Similarly, this hydrogen binding network is predominantly conserved within close evolutionary homologs serine dehydratase (SDH) and threonine deaminase. By aligning 186 sequences, Mozzarelli and co-workers found that the T52 position showed the highest variability while Q89 is conserved in enzymes that are allosterically regulated by nucleotides (i.e., hSR, spSR, threonine deaminases) (Canosa et al., 2018). On the other hand, *Hordeum vulgare* SR and SDH have either an alanine or methionine at this position and are not regulated by ATP.

Upon mutation of the Q89 residue to either a methionine or an alanine, ATP activation is reduced from a 7-fold increase in the wt-enzyme to only 4-fold and 2-fold for the Q89M and Q89A mutants, respectively (Canosa et al., 2018). Moreover, this effect is not due to the decrease in ATP affinity, as addition of ATP at higher concentrations still fails to show activation in the mutants. Interestingly, the Q89 mutants maintain the same activity as the wt-enzyme in the absence of ATP. Studies show that these mutants exhibit non-cooperative binding with respect to ATP. This can also be observed in the crystal structures solved to date in which two different conformations of Q89 have been observed (Figure 4). It is postulated that this residue acts as a key gating residue, playing a central role in the conformational change associated with allosteric activation of the enzyme (Canosa et al., 2018).

## Pyridoxal Phosphate Site

### Phosphate Binding Pocket

**Figure 5:**
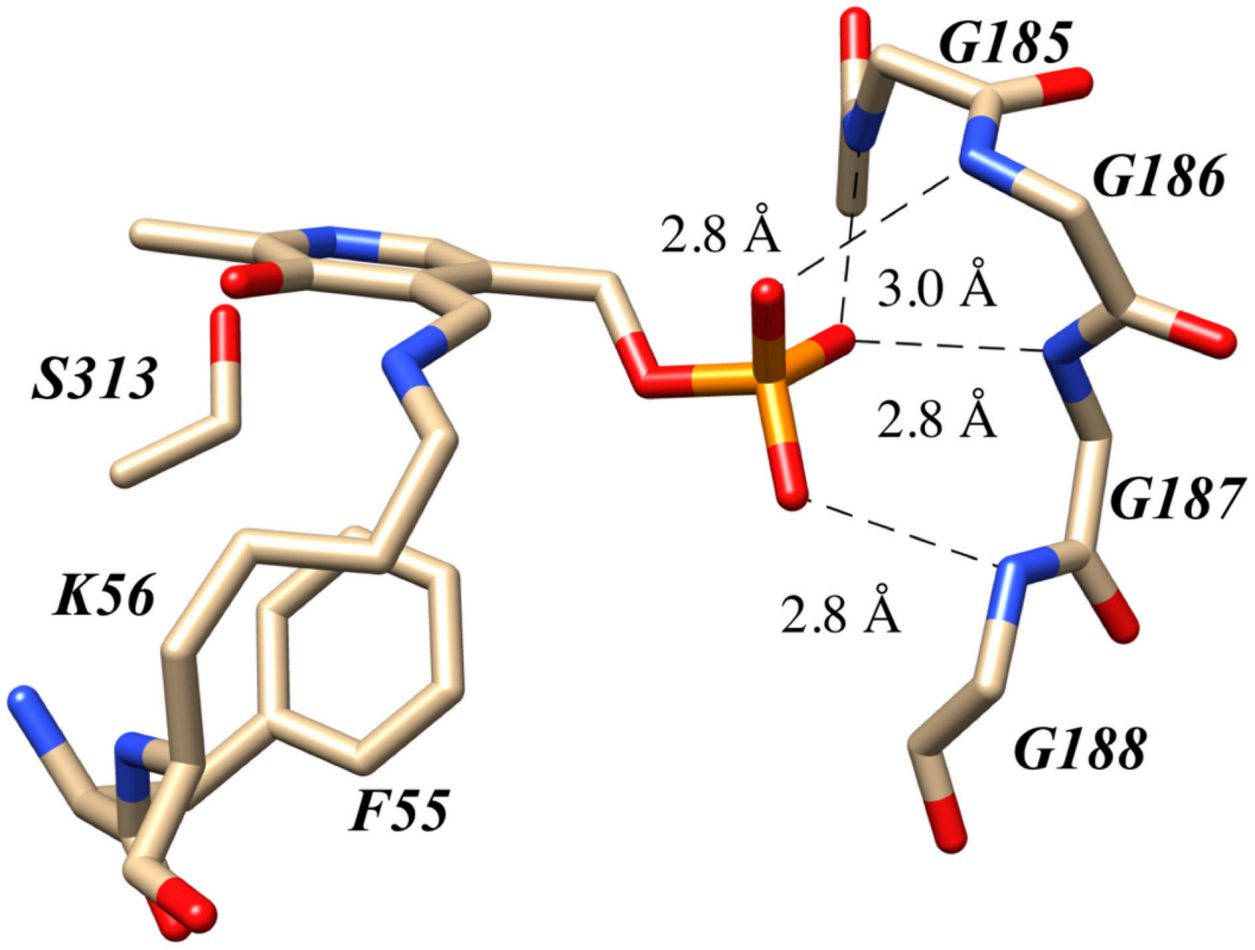
The canonical tetraglycine loop that serves as a PLP phosphate binding pocket as seen in the hSR structure PDB code:3L6B.

**Figure 6:**
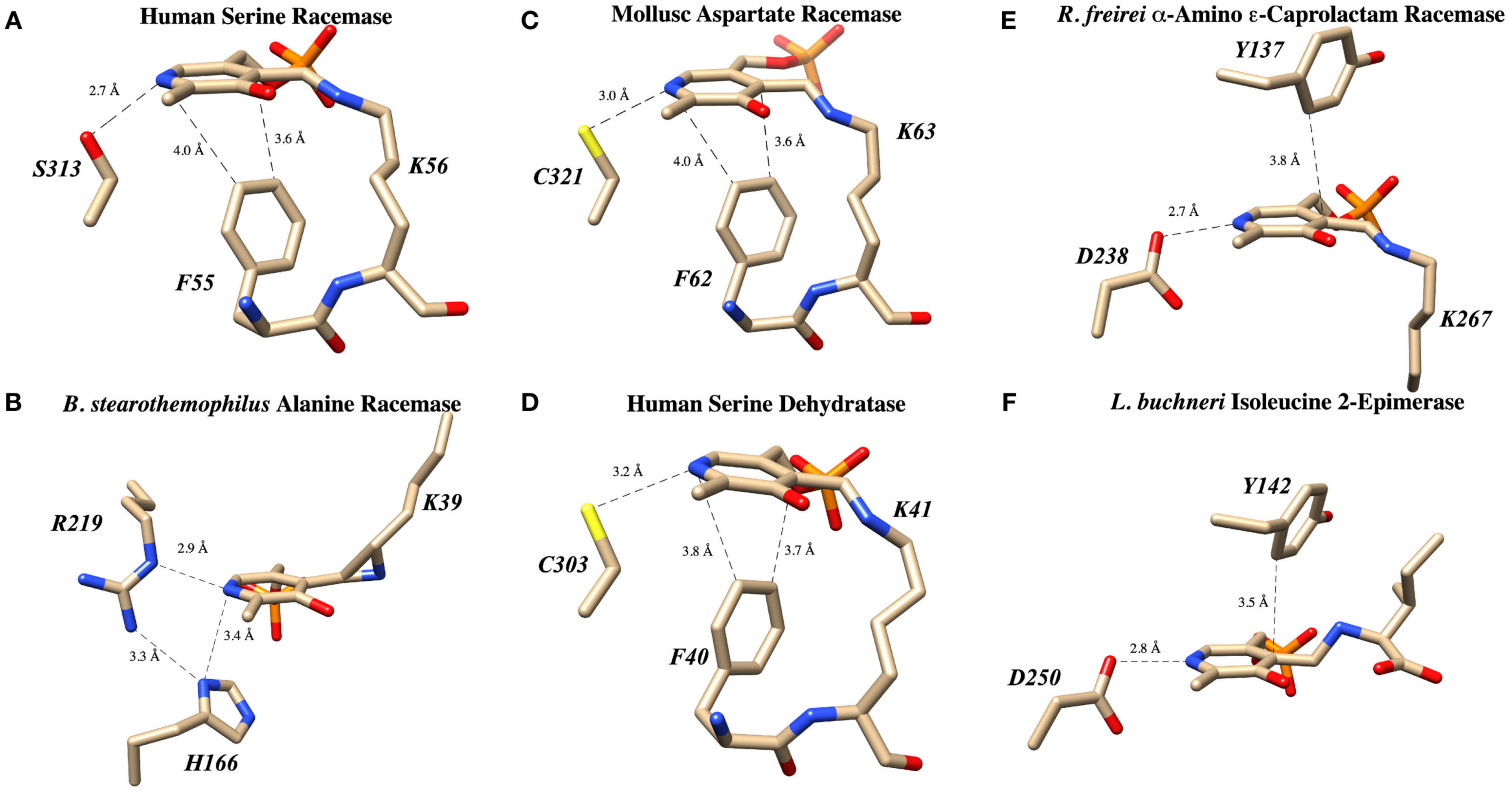
PLP binding sites in known racemase structures illustrating hydrogen bonding and π-facial interactions of the pyridine ring. **(A)** hSR: pyridine H-bonding with S313 and edge-to-face π-π interaction with F55 (PDB code: 3L6B). **(B)** Alanine racemase: presumably weak pyridine H-bonding with R219 (PDB code: 1SFT). **(C)** Aspartate racemase: pyridine H-bonding with C321 and edge-to-face π-π interaction with F62 (PDB code: 5YBW). **(D)** Human serine dehydratase: pyridine H-bonding with C303 and edge-to-face π-π interaction with F40 (PDB code: 4H27). **(E)** α-Amino ε-caprolactam racemase: pyridine H-bonding with D238 and edge-to-face π-π interaction with Y137 (PDB code: 5M46). **(F)** Isoleucine 2-epimerase: pyridine H-bonding with D250 and edge-to-face π-π interaction with Y142 PDB code: 5WYA.

Human serine racemase displays a classical PLP binding site, including all the hallmark attributes as follows: (i) the tetraglycine loop for binding of the 5′-phosphate (Smith et al., 2010), (ii) π-stacking interaction to engage the pyridine ring (Smith et al., 2010), and (iii) hydrogen-bonding to the pyridine-nitrogen. The tetraglycine loop for hSR consists of a string of glycines from position 185 to 188, each utilizing an amide N-H to donate a hydrogen bond for phosphate binding (Smith et al., 2010). This canonical PLP enzyme feature is present in most cofactor binding sites and is quite evident in the hSR structure (Figure 5). While the PLP binding site is highly conserved across most PLP enzymes (see Figure 6) it is important to note that PLP-dependent enzymes fall into a wide range of fold types, which has been discussed nicely elsewhere (Schneider et al., 2000). For our purposes here, it is notable that PLP-dependent racemases themselves fall into more than 1-fold type, with serine racemase (Yoshimura and Ito, 2014) and aspartate racemase (Takahashi, 2009) being members of the fold type II family and alanine racemase being a fold type III enzyme (Azam and Jayaram, 2016).

### π-Stacking

The second key stabilizing feature often found in PLP cofactor binding sites is an aromatic amino acid side chain engaged in a favorable π-π interaction with the pyridine ring. In enzymes such as serine racemase (Figure 6A) (Smith et al., 2010), aspartate racemase (Mizobuchi et al., 2017) (Figure 6C) and serine dehydratase (Figure 6D) (Wang et al., 2012), the aromatic ring for π-stacking is provided by a Phe residue immediately preceding the essential lysine in the primary sequence. The aromatic ring of this Phe side chain is engaged in an edge-to-face π-π-interaction at the *si*-face of the PLP-ring. Other racemases, such as α-amino ε-caprolactam racemase (Figure 6E) (Frese et al., 2017) and isoleucine 2-epimerase (Figure 6F) (Hayashi et al., 2017), utilize a tyrosine side chain for a similar edge-to-face interaction.

### Pyridine Nitrogen

The third feature of most PLP-binding pockets is the presence of a hydrogen bond-donating side chain that partially protonates the pyridine nitrogen in the ground state. For transaminase enzymes, this residue is generally an aspartate residue (Chan-Huot et al., 2013; Fesko et al., 2018), presumably promoting formation of a quinonoid intermediate with broad charge delocalization. It has been argued that the intermediacy of such a charge-delocalized species facilitates the required azallylic isomerization (i.e., C4'-protonation) for such enzymes. For racemase enzymes, however, it is clear that such an acidic proton donor is not required. For example, perhaps the most well-studied PLP-dependent racemase, alanine racemase, utilizes an arginine residue in this position, a weak hydrogen bond donor (Figure 6B) (Shaw et al., 1997).

A survey of current PLP-dependent racemase structures in the pdb shows that, in fact, these enzymes feature a broad range of proton donors for the pyridine ring. In aspartate racemase (Mizobuchi et al., 2017), Cys321 serves as H-bond donor (Figure 6C-PDB code: 5YBW), whereas α-amino ε-caprolactam racemase (Figure 6E-PDB code: 5M46), (Frese et al., 2017) and isoleucine 2-epimerase (Figure 6F-PDB code: 5WYA) (Hayashi et al., 2017) utilize aspartatic acid residues Asp238 and Asp250, respectively, as PLP-nitrogen protonating residues. Serine racemase (Figure 6A–PDB code: 3L6B) (Smith et al., 2010) employs a serine residue, Ser313, reminiscent of β-eliminase enzymes such as tryptophan synthase or O-acetylserine sulfhydrylase (OASS). This is interesting because SR, like tryptophan synthase, catalyzes the β-elimination of water from L-serine. That said, not all β-eliminase enzymes employ a serine residue, as serine dehydratase utilizes a cysteine (Figure 6D–PDB code: 4H27) (Wang et al., 2012).

The greater need for charge delocalization (and hence pyridine N-protonation) for PLP-enzyme-mediated transamination, as opposed to β-elimination or racemization chemistry, is supported by the results of a seminal study employing deaza-PLP (Griswold and Toney, 2011). These workers compared all three of these classes of PLP enzymes with both the native cofactor and its synthetic deaza-analog. Upon removing the ring nitrogen, by far the biggest penalty paid in k_cat_ is for transamination, with aspartate aminotransferase suffering an ~10^9^-fold decrease in activity. On the other hand, the β-eliminase OASS experiences only a 260-fold decrease and alanine racemase sees an ~700-fold decrease in k_cat_. Griswald and Toney conclude that upon breaking the Cα-H bond, transaminases delocalize charge fully into the PLP ring, while β-eliminases and racemase enzymes operate via a “carbanionic intermediate” with a more localized azallylic charge distribution across Cα and C4'.

### Nature of the Electron Sink: Quinonoid vs. Carbanionic Intermediate

The PLP-imine π-system is often described as a four-electron sink. The pyridine nitrogen is thought of as the primary locus for the first two electrons stored in the extended π-system, with the imine nitrogen then in position to accept a second pair of electrons as, for example, would be required for a γ-replacement enzyme. Prior to such elegant physical organic chemical tools that are now available to interrogate reaction mechanism, it was long assumed, following the pioneering stereoelectronic arguments of Dunathan (Dunathan, 1966), that PLP enzymes catalyzing Cα-H, Cα-C, or Cα-COOH bond cleavage would fully delocalize the resultant electron density into the π-system of the PLP-imine, the most stable resonance form of which would place those electrons on the pyridine nitrogen (Walsh, 1979). The ability to form a charge-balanced, net-neutral quinonoid intermediate would then require the pyridine nitrogen to be protonated. While this is clearly possible when an Asp (or potentially Glu) residue is so positioned in the active site, full protonation of the PLP ring nitrogen with a Ser, Cys, or Arg residue, for example, would require that these residues have abnormally low pK_a_ values in the given PLP enzyme active site.

Consistent with this reasoning, to our knowledge, quinonoid intermediates have only been observed for PLP enzyme active sites that do feature an acidic residue donating a proton to the PLP ring nitrogen. Because such quinonoid intermediates feature an extended quinone-like π-system, these species absorb well into the visible, typically with λ_max_ ~ 480–550 nm. Quinonoid intermediates have been observed by stopped flow spectrophotometry in enzymes that natively feature aspartate residues protonating the pyridine nitrogen (Metzler et al., 1988; Phillips et al., 1998; Karsten et al., 2005). In enzymes in which this residue is natively a serine, such as tryptophan synthase (Jhee et al., 1998) or an arginine such as alanine racemase (Sun and Toney, 1999), mutation of these residues to Asp or Glu, respectively, allows for the observation of quinonoid intermediates that had otherwise been unobservable.

**Figure 7:**
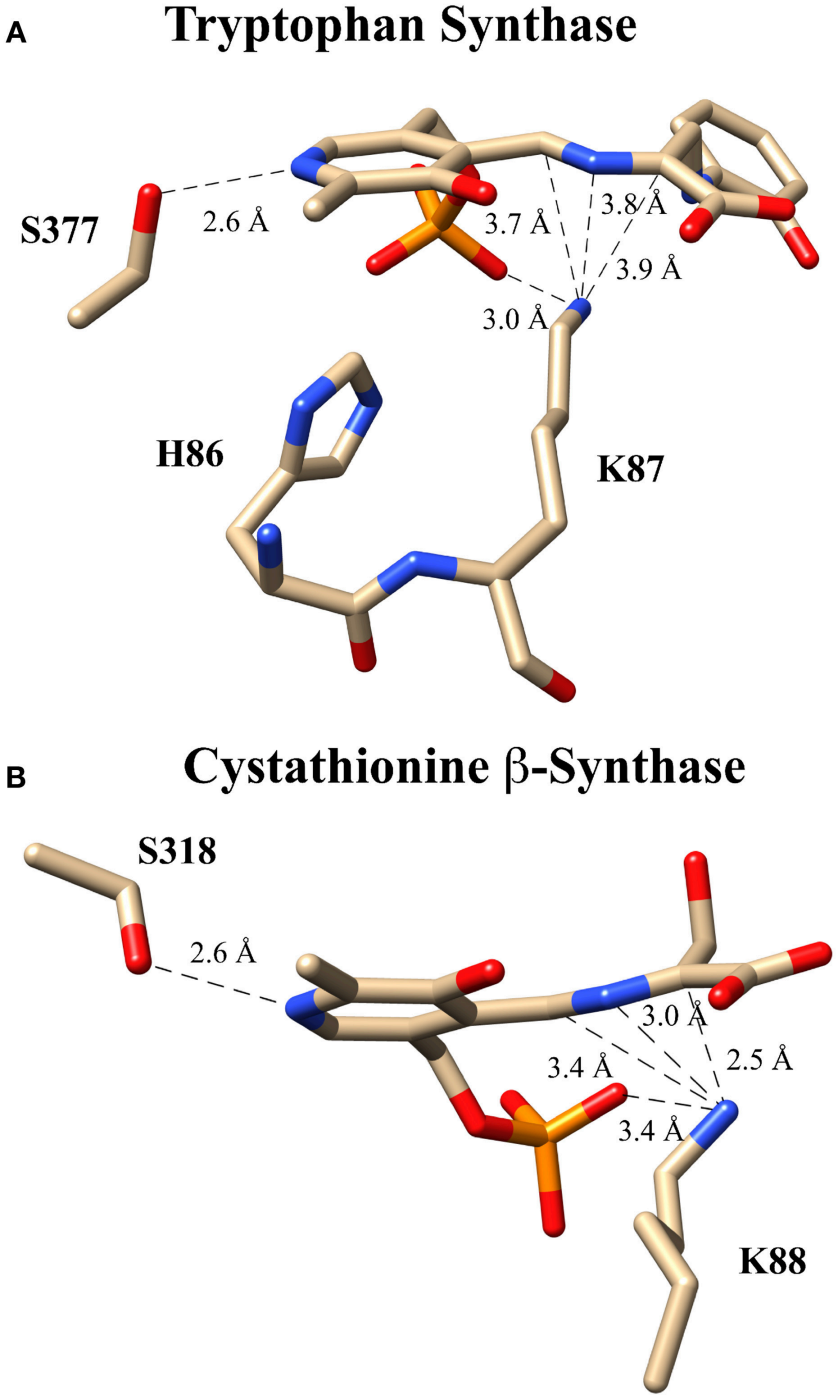
Putative carbanionic intermediates as observed via x-ray crystallography. **(A)** Tryptophan synthase structure evidencing a “carbanonic intermediate” formed by addition of 2-aminophenol to the aminoacrylate intermediate (PDB code: 4HJP). This model is supported by Gaussian 09-based computations. **(B)** Cystathionine β-synthase structure featuring a similar “carbanionic intermediate” observed at pH 7 and hypothesized to be electrostatically stabilized by K88 (PDB code: 3PC4).

These experiments suggest several alternative possibilities for catalysis with an enzyme such as tryptophan synthase. Catalysis might proceed (i) in a concerted fashion without buildup of negative charge in an intermediate or transition state, (ii) via a more localized carbanionic intermediate in which the charge is not delocalized significantly into the aromatic π-system of the PLP ring, or (iii) via a fully delocalized quinonoid intermediate with a lifetime that is too short to observe with typical stopped flow instruments. In fact, collaborative work by Dunn and Mueller, utilizing a combination of NMR, X-ray crystallography and computational modeling (Caulkins et al., 2016; Huang et al., 2016), provides evidence for the intermediate case just described; namely, for the formation of such a localized “carbanionic intermediate.” As is shown in Figure 7A, this non-planar intermediate is thought to distribute electron density across the Cα-N-C4'-azallylic system rather than into the pyridine π-system. The active site lysine ε-ammonium ion is seen in close enough proximity to electrostatically stabilize this “carbanionic intermediate.”

A related observation was made for the enzyme drosophila cystathionine β-synthase (CBS), by Banerjee, Smith and co-workers via x-ray crystallography (Figure 7B) (Koutmos et al., 2010). Here, too, a non-planar structure is seen for the putative “carbanionic intermediate” with L-serine substrate at pH 7. The azallylic C4'-N-Cα-anion is puckered upward toward the *re*-face, out of the plane of the pyridine ring system. Upon lowering the pH to 6.5, β-elimination apparently occurs, and one sees the resultant aminoacrylate intermediate in the crystal. Consistent with these crystallographic results, stopped flow spectrophotometric analysis provides evidence for an aminoacrylate intermediate at 460 nm. Another intermediate is also seen at 315 nm, potentially the azallylic carbanionic species, as such a lower λ_max_ value would be expected for such a system with limited charge delocalization.

It should be noted that both cystathionine β-synthase and tryptophan synthase have similar active sites to that of serine racemase, as all three contain a serine hydroxyl in position to protonate the pyridine ring nitrogen. These studies thus suggest that the bifurcating racemase/β-eliminase activity of SR may proceed by way of such an incompletely delocalized “carbanionic intermediate.”

## Active Site

### Essential Lysine

The essential lysine residue is found in all PLP-dependent enzymes, serving as a handle for the covalent attachment of the PLP cofactor in the internal aldimine. There are clear kinetic advantages of such an aldimine linkage, as this allows the amino acid substrate to rapidly form the external aldimine via a facile transaldimination reaction that passes through a *gem*-diamine intermediate. Essential lysine mutants generally lose orders of magnitude in catalytic efficiency but are still useful for the study of enzyme structure and mechanism. These mutants often retain the ability to non-covalently bind the PLP cofactor and support formation of the external aldimine by a much less efficient amine-aldehyde condensation rather than by the usual transaldimination mechanism. Perhaps, more importantly, such a mutation also means that the *si*-face base has been lost. For example, in possibly the most well-studied PLP enzyme, aspartate aminotransferase, the K258A mutant exhibits a 10^8^-fold decrease in activity (Toney and Kirsch, 1993; Griswold and Toney, 2011) relative to the wild type. In CBS, the K119A mutant displays a 10^3^-fold decrease in activity. However, addition of the exogenous base ethylamine allows for a 2-fold gain in activity, perhaps reflecting external compensation for this lost *si-*face base activity (Evande et al., 2004).

### Putative *re-*face Base

In hSR, the essential lysine, Lys56, is thought to be the *si*-face base responsible for α-deprotonation of L-serine or related substrates in human serine racemase. Ser84 has been proposed to be the complementary *re*-face base, responsible for reprotonation at the α-carbon to form D-serine. Crystallography demonstrates that this serine is highly conserved for hSR and its homologs in various other organisms (Goto et al., 2009; Koutmos et al., 2010). However, at physiological pH, the serine hydroxyl pK_a_ is estimated to be too high to serve as a catalytically efficient general acid. Recently, based upon crystallographic considerations, Berkowitz and co-workers suggested a possible mechanism through which the effective pK_a_ of Ser84 may be lowered in the hSR active site. Namely, these workers noted that the hSR structure features a potential Ser84-Wat372-Lys114 hydrogen bonding network (Nelson et al., 2017) resembling the Ser-*cis*-Ser-Lys catalytic triad of the amidase signature enzyme family (Ekici et al., 2008; Mileni et al., 2009; Pratt and McLeish, 2010; Lee et al., 2015; Cerqueira et al., 2017).

Several groups have expressed the *re*-face base Ser to Ala mutant in serine racemase enzymes from *Dictyostelium discoideum* (slime mold), *S. pombe*, and humans (Goto et al., 2009; Bodhinathan et al., 2010; Nelson et al., 2017). In all cases, as expected, racemization activity is lost. For the mammalian enzyme, β-elimination of L-serine to pyruvate persists but undergoes a 6-fold reduction in catalytic efficiency (k_cat_/K_m_), as can be seen in Table 1. The normal hSR preference for the negatively charged β-elimination substrates L-threo-β-hydroxy-aspartate (L-THA) and L-serine-*O*-sulfate (L-SOS), also persists in the S84A mutant (Strísovský et al., 2005).

**Table 1:** Kinetics of hSR mutants highlighting the S84D mutants switch in preference to elimination of serine over charged substrates and the interesting preference of S84T for L-SOS over L-THA [table adapted with permission of the American Society for Biochemistry and Molecular Biology (ASBMB) (Nelson et al., 2017)].

	**k**_**cat**_**/K**_**m**_ **(Sub.)**: **k**_**cat**_**/K**_**m**_ **(L-Ser)**		
**Variant**	**L-SOS**	**L-THA**	**L-Ser vs. L-SOS vs. L-THA**
WT	100:1	93:1	1:100:93
S84A	71:1	34:1	1:71:34
S84D	1:12	1:50	50:4:1
S84N	2.5:1	7:1	1:2.5:7
S84T	370:1	50:1	1:370:50

### S84D Mutant Reveals Importance of R135 in Controlling β-Elimination Substrate Preferences

When Ser84 is mutated to an acidic aspartate residue, the S84D mutant again loses the ability to catalyze the racemization reaction, as expected. The β-elimination chemistry of this mutant, however, demonstrates a surprising reversal of substrate preference. The native enzyme prefers the elimination substrates L-SOS and L-THA, each of which displays a negatively charged side chain over L-Ser ~100:1. This ratio changes to 50:1 in favor of L-Ser in the S84D mutant. This corresponds to a ~5,000-fold swing in L-Ser to L-THA preference and a ~1,200-fold change in L-Ser to L-SOS processing efficiency. The S84D hSR mutant thus displays a an inverted β-elimination substrate bias toward L-Ser of 50:1 vs. L-THA and of 12:1 vs. L-SOS (Nelson et al., 2017).

**Figure 8:**
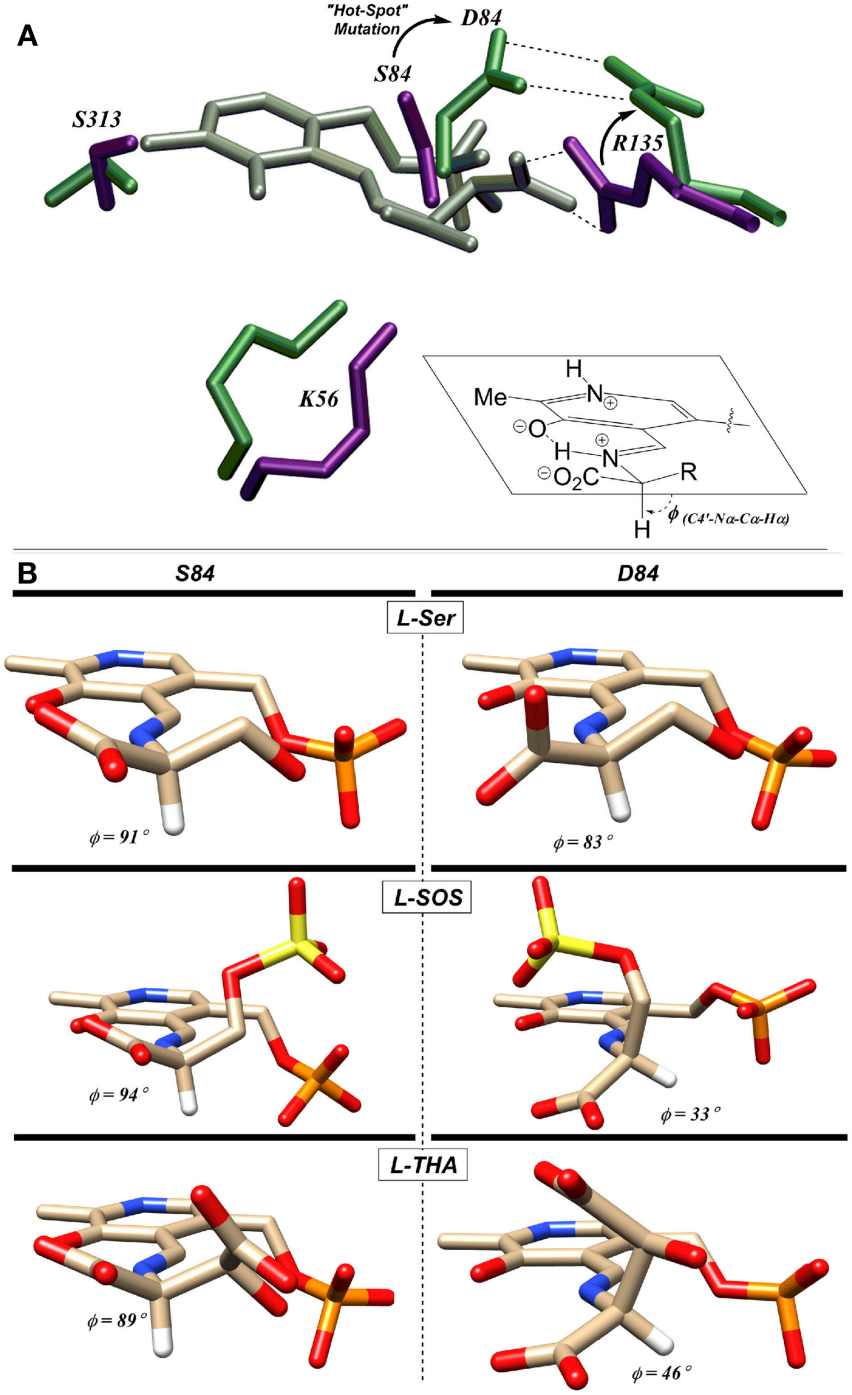
**(A)** L-THA-derived external aldimine (gray) docked with native-hSR (violet) and S84D hSR (green). The putative L-THA-R135 salt bridge is replaced with a D84-R135 salt bridge in the mutant. **(B)** Docked external aldimines of L-Ser, L-SOS, and L-THA exhibiting much better Dunathan alignment in wt-hSR (left) than in S84D-hSR (right) [Figure adapted with permission of the American Society for Biochemistry and Molecular Biology (ASBMB) (Schell, 2004; Nelson et al., 2017)].

Utilizing molecular dynamics simulation and docking, the Berkowitz group put forth a model based upon the Dunathan hypothesis (Dunathan, 1966) that is consistent with this finding (Nelson et al., 2017). The model is based upon stereoelectronics and the notion that the Cα-H bond to be broken must be aligned with the extended π-system of the PLP-imine (Dunathan, 1966). For the wild-type enzyme, negatively charged substrates are predicted to be oriented via a salt bridge with R135 resulting in the proper alignment for deprotonation (Figure 8A). This model is also consistent with the crystal structure of hSR bound to malonate (3L6B), in which the β-carboxylate of the inhibitor forms a salt bridge with R135 (Koutmos et al., 2010). Molecular dynamics simulations of the S84D mutant suggest that D84 moves to form a new salt bridge with R135, thereby preventing the positively charged arginine guanidinium group from interacting with the negatively charged side chains of L-THA and L-SOS. This results in a less-than-optimal positioning of these substrates in their respective enzyme-bound external aldimines, with the Dunathan angle (dihedral angle = H-Cα-N-C4′) distorted from the ideal 90–46° and 33° for L-THA and L-SOS, respectively (Figure 8B) (Nelson et al., 2017).

In light of the S84D results, the S84N hSR mutant was also studied. Intermediate results were observed, with only a modest preference for L-SOS (2.5:1) and L-THA (7:1) over L-Ser being observed. L-Serine racemization was not detected but the β-elimination was nearly at wild-type catalytic efficiency. Molecular modeling suggests that the intermediate activity seen with L-THA may be due to two different conformations of the hSR-bound external aldimine; in one, the R135 guanidinium group is engaged with the β-carboxylate of the substrate (better Dunathan alignment ~ 82°), and in the other, R135 forms a salt bridge with the α-carboxylate (Nelson et al., 2017).

Finally, the S84T mutant was also studied. This is the only hSR mutant studied that retains L-Ser racemization activity, albeit with a 10-fold loss in efficiency. The β-elimination reaction of L-Ser is also less efficient by ~1 order of magnitude. As for the β-elimination reaction of the negatively charged substrates, this mutant shows a marked preference for L-SOS elimination (370:1 vs. L-Ser) over L-THA (50:1 vs. L-Ser) elimination. This L-SOS preference may be due to the fact that this substrate features a favorable β-sulfate leaving group that need not be protonated to leave, as compared with L-THA, for which the β-OH leaving group would require protonation to leave. It may simply be that in the S84T mutant, the general acid required for β-OH protonation in L-THA is not optimally positioned to do so (Nelson et al., 2017).

### Lys-N^ε^-Ala57 Extender Arm Variant

An interesting apparent self-catalyzed active site modification reaction has been reported for the serine racemase from *Schizosaccharomyces pombe* (SpSR) by Esaki and Hirotsu and co-workers (Goto et al., 2009; Yamauchi et al., 2009). In one of the first three crystal structures published by this group for the *S. pombe* SR, it was observed that the essential lysine had been modified to a L-lysino-D-alanyl-residue upon extended incubation with L-serine. This amounts to a three-atom extension of the active site Lys-57 residue. The authors provide both mass spectrometric (+87) and x-ray crystallographic evidence in support of this structure. They claim that a 97% level of modification is seen.

**Figure 9:**
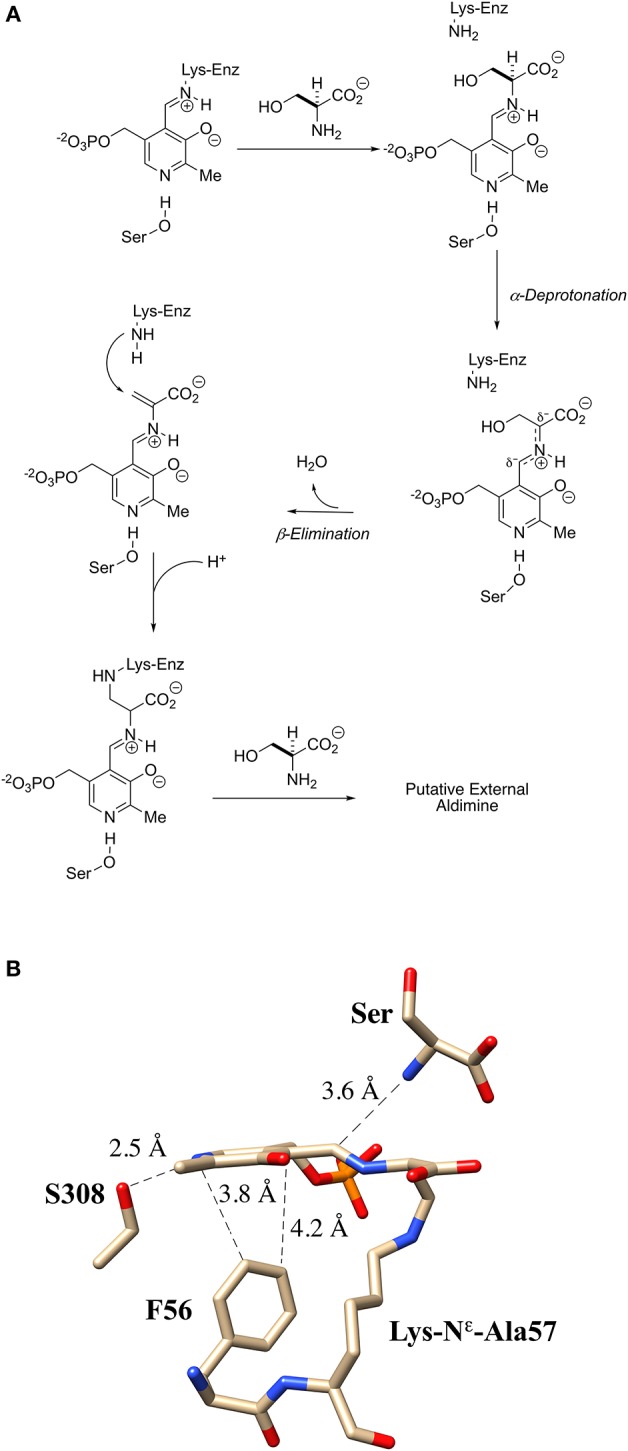
Extender arm of the essential lysine observed by crystallography of *S. pombe* serine racemase. **(A)** Potential mechanism for formation of the extender arm. **(B)** Crystal structure of the internal aldimine with the “extended”-lysine residue (2ZR8).

Even though this modification places a carboxylate group alpha- to this active site “extended” lysine residue, the modified SpSR maintains 54% of the racemization activity and 68% of the β-elimination activity of the wt-enzyme. This self-catalyzed modification of the essential lysine of SpSR is proposed to occur by elimination of water from serine and the conjugate addition of the essential lysine into the resultant PLP-bound aminoacrylate intermediate (Figure 9A). In addition to revealing the three-dimensional structure of this modified enzyme, the crystal structure also appears to show a near attack conformation of an L-serine molecule approaching the lysino-D-alanyl-internal aldimine as would be expected for a transaldimination reaction of this modified enzyme (Figure 9B) (Goto et al., 2009). It remains to be seen if similar behavior will be observable in mammalian SR enzymes.

### Residues Influencing Racemization vs. β-Elimination—“Triple Serine Loop”

As is illustrated schematically in Figure 2, one observes a bifurcating L-Ser racemization (giving D-Ser product) vs. L-Ser β-elimination (giving pyruvate product) mechanism for the enzyme serine racemase. This raises several important questions. (1) Is this observed bifurcating activity biologically relevant, incidental, or even an artefact of the assay conditions *in vitro?* (2) If the bifurcation is biologically relevant, what are the key biological determinants of whether hSR drives L-Ser substrate more toward D-Ser or pyruvate? (3) What are the key structural/mechanistic features along the reaction coordinate that control the “decision” to racemize or β-eliminate substrate?

As to the relevance of the *in vitro* assay, it is necessary to note here that hSR is known to be engaged in protein-protein interactions *in vivo* that may influence catalytic activity and/or bifurcation ratio. These include reported interactions with PICK 1 (protein interacting with C-kinase) (Hikida et al., 2008), GRIP (glutamate receptor interacting protein) (Baumgart et al., 2007), stargazin and PSD95 (postsynaptic D protein 95) (Ma et al., 2014) and DISC 1 (disrupted in schizophrenia) (Ma et al., 2013; Xia et al., 2016). That said, for now, in the absence of compelling evidence that the overall kinetic profile is significantly altered by such PPIs, we will proceed to analyze hSR performance by steady-state kinetic analysis in isolated enzyme assays, with both divalent cation and ATP present.

As we and others have noted (Strísovský et al., 2003; Nelson et al., 2017), under such conditions, wt-hSR favors the L-Ser β-elimination reaction over the racemization reaction by a 4-5:1 ratio. It may be that the β-elimination reaction serves as a sort of “bleed valve,” potentially allowing local stores of L-Ser to be diverted to pyruvate and away from D-Ser as a mechanism for muting D-Ser signaling. This could be a sort of secondary checkpoint, providing a mechanism for managing steady-state L-Ser levels in the neuron, beyond the control that is exercised by the rate at which L-Ser is produced from 3-phosphoglycerate in the astroglia and shuttled to the neuron (Figure 1) (Ishiwata et al., 2015; Wolosker et al., 2016). There has been a related discussion on hSR-mediated D-Ser β-elimination being a mechanism for controlling D-Ser concentrations in the neuron (Foltyn et al., 2005; Wolosker, 2011).

As is illustrated in Figure 2, probably the most streamlined mechanism for this bifurcation would involve initial external aldimine formation of L-Ser, followed by *si*-face deprotonation by K56 to a common “carbanionic” or quinonoid intermediate. As has been discussed earlier, elegant model studies by the Toney group (Griswold and Toney, 2011) suggest that neither racemase nor eliminase activity requires a completely delocalized quinonoid intermediate. These observations are consistent with the observation of “carbanionic intermediates” for the β-eliminase/replacement enzymes, CBS (Koutmos et al., 2010) and tryptophan synthase (Caulkins et al., 2016), as noted in Figure 7. As is shown in Figure 2, a likely decision point for bifurcation would then occur at the protonation step, with *re*-face protonation, presumably by Ser84, leading to racemization and with OH-leaving group (LG) protonation leading to β-elimination.

**Figure 10:**
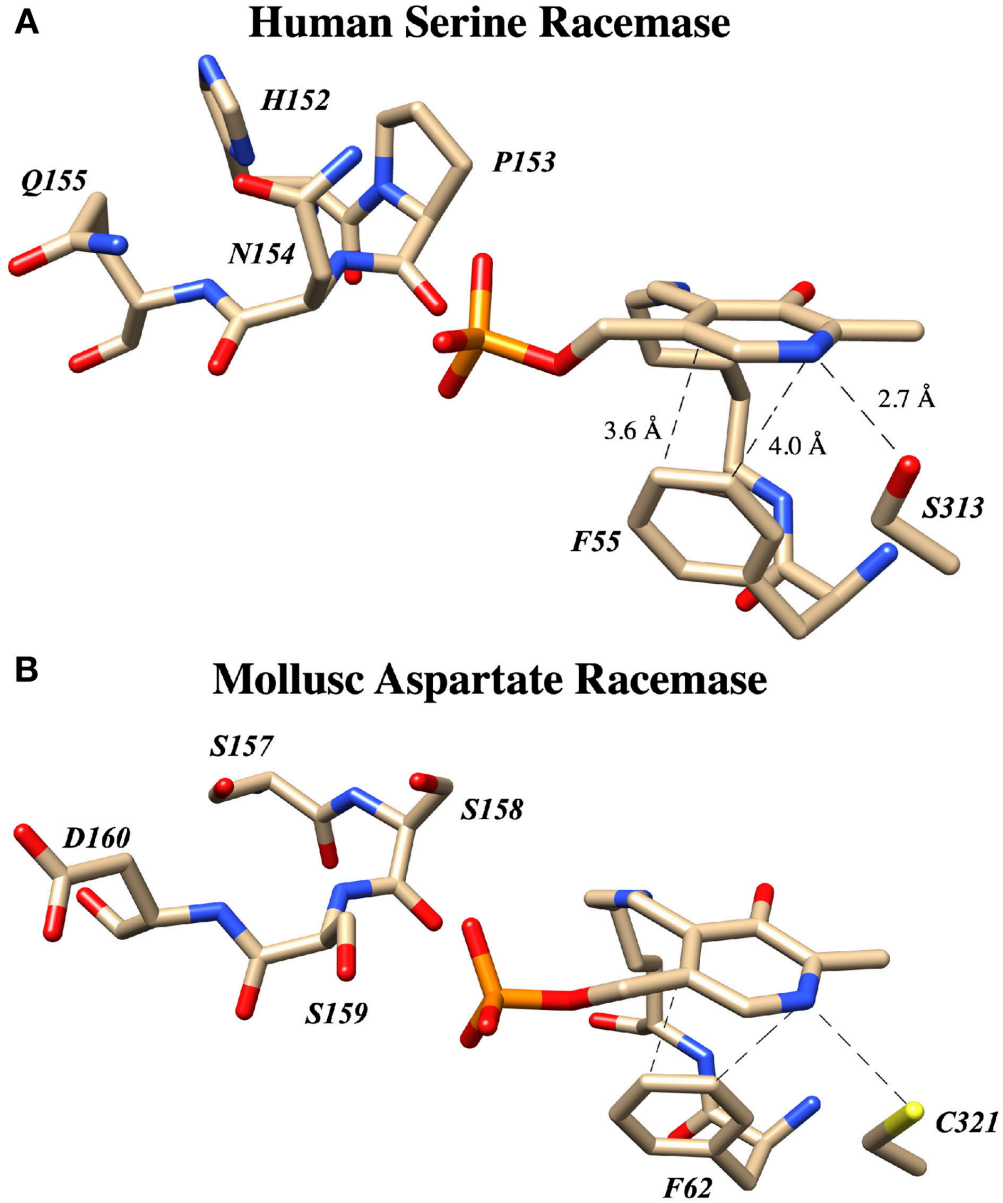
The “Triple serine loop” in hSR and *Anadara broughtonii* (AbAR) is situated near the presumed locus of the external aldimine and the carbanionic intermediate derived therefrom. **(A)** Crystal structure of human serine racemase (3L6B) loop region containing H152, P153, N154, and Q155. **(B)** Corresponding region of aspartate racemase (5YBW) with residues S157, S158, S159, D160.

Both the groups of Toney (Foltyn et al., 2005) and of Uda et al. (2016, 2017) have reported studies demonstrating that mutation of targeted residues can profoundly influence the racemization to β-elimination ratio in hSR and homologs. These results are summarized in detail in Table 2. Uda et al. performed a detailed phylogenetic analysis of the serine/aspartate racemase family and deduced that a so-called “triple serine loop” (Figure 10; named after the wt-AR sequence) may be critical for racemization function as residues here appear to correlate with a likely evolution from SR to AR activity.

**Table 2:** Kinetic indication that mutations of SR residues in the “triple serine loop” to the corresponding aspartate racemase (AR) residues tends to bias enzyme activity toward racemization over β-elimination.

**Variant**	**Position**				**Racemization Efficiency^‡^**	**$k_{cat}(rac):k_{cat}(\text{β}-elim)$**	$K_{cat}/K_{m}(rac):k_{\;cat}/K_{m}(\text{β}-elim)$	**References**
	$\overset{\;}{\overset{︷}{\begin{array}{cccc} 152 & 153 & 154 & 155 \end{array}}}$							
**“Triple Ser Loop”- Relation to Racemase Activity**								
hSR^+^	H	P	N	Q	100	1:4	1:3.7	Hoffman et al., 2009; Nelson et al., 2017; Canosa et al., 2018
mSR^+^	H	P	N	Q	133	1:2.3	1:1.3	Uda et al., 2016
mSR	S	P	N	Q	–	–	1:1.4	Foltyn et al., 2005
mSR	S	P	N	Q	121	rac only*	rac only*	Uda et al., 2017
mSR	H	S	N	Q	–	–	**4:1**	Foltyn et al., 2005
mSR	H	S	N	Q	1140	**23:1**	**2:1**	Uda et al., 2017
mSR	H	P	S	Q	174	**6.8:1**	**2.3:1**	Uda et al., 2017
mSR	H	P	N	D	46 (700)^‡^	**7:1**	**7.3:1**	Foltyn et al., 2005
mSR	S	S	N	Q	100	rac only*	rac only*	Uda et al., 2017
mSR	H	S	S	Q	376	**3.5:1**	1:12.8	Uda et al., 2017
mSR	S	P	S	Q	160	rac only*	rac only*	Uda et al., 2017
mSR	S	S	S	Q	55	rac only*	rac only*	Uda et al., 2017
spSR^+^	P	P	Y	D	**	1:29^¶^	1:26^§^	Yamauchi et al., 2009
amAR^+^	H	S	S	D	24	**8.6:1**	**2.2:1**	Uda et al., 2017
cgAR^+^	S	S	S	D	18	rac only*	rac only*	Uda et al., 2017

*Residue numbering corresponds to the hSR sequence. SR wild type residues are in **blue**; AR from Acropora millepora (amAR) and Crassostrea gigas (CgAR) are being compared here with the CgAR wild type residues appearing in **green**. Emboldened entries indicate enzymes for which racemization is preferred over elimination*.

+ *designates the wild type enzyme*.

‡ *These values are normalized to the average value of k_cat_ for racemization for wt hSR (30 ± 15 min^-1^) which is arbitrarily set at 100*.

* *no β-elimination activity observed*.

*the value in parentheses here represents the value of kcat(rac) for this mutant normalized to the k_cat_(rac) of the wt-mSR reported by these authors which is more than an order of magnitude lower than that determined by other groups*.

** these authors report “V_max_ = 30 U/mg,” but this is actually a specific activity reported for a standard assay ^@^ [L-Ser] = 10 mM. Since this concentration is very close to K_m_ for L-Ser, the velocities reported are well-below V_max_.

¶ *this value represents relative velocity for racemization vs. elimination at 10 mM concentration rather than a ratio of kcat values*.

§ *whereas the K_m_ values for these activities are reported, k_cat_(V_max_) values are not. For the latter, reported relative velocity for racemization vs. elimination at 10 mM concentration is given*.

Specifically, L-THA dehydratases and SRs from lower order organisms possess a loop region from amino acids P150, P151, and Y152. Tracing this loop region up the phylogenetic tree demonstrates that these residues change to H152, P153, and N154 in human and mouse SR. On the other hand, in aspartate racemases, these residues morph into a “triple serine loop” of sequence S150, S151, S152 as for example in the AR from *Crassostrea gigas* (CgAR) (Uda et al., 2016). For CgAR, this SSS motif appears to dictate the substrate preference for L-Asp over L-Ser. Wild-type *S. pombe* SR with the PPY sequence exhibits no AR activity, and mouse SR with the MPN sequence shows limited AR activity with k_cat_/K_m_ ~ 65 mM^−1^min^−1^ (Bodhinathan et al., 2010; Uda et al., 2016).

A secondary finding from these studies is that, in general, introducing residues representing the AR sequence into this loop in hSR tends to favor L-Ser racemization, by a combination of suppressing L-Ser β-elimination (or dehydrase activity) and promoting L-Ser racemization. Uda and co-workers cloned nearly a dozen SR and aspartate racemase enzymes and studied both the wt- and mutant versions of these enzymes kinetically. Earlier, the Toney group had also looked at SR mutants in this loop. From Table 2, it can be seen that installation of S residues at positions 153 and 154 in hSR in particular tends to increase the k_cat_ for racemization. Introduction of S into position 152 in mSR significantly decreases L-Ser β-elimination activity; a similar effect appears to result from introduction of a D residue into position 155.

Most importantly, these studies demonstrate that for hSR, the bifurcation ratio is controlled significantly at the level of the primary sequence, with particular sensitivity to modifications in this “triple serine loop.” Figure 10 illustrates that this loop is located just above the PLP-imine functionality in the external aldimine crystal structures for both the SR and AR enzymes. To understand the molecular basis for how specific mutants morph hSR activity from the native predilection for β-elimination to a preference for racemization, the tools of structural biology could be of great value.

## Discussion and Concluding Remarks

From an evolutionary standpoint, it appears that serine racemase activity may have evolved from L-*threo-*hydroxyaspartate (L-THA) eliminase activity and may also have served as the evolutionary precursor to aspartate racemase (AR). Consistent with this view, both the *re*-face base S84 and R135 in the human isoform are highly conserved across the SR family, and these residues are also conserved among enzymes demonstrating L-THA dehydratase activity (Nelson et al., 2017). Additionally, modifications in the loop region corresponding to positions 152–155 in hSR, from HPNQ to SSSD, appear to have been primary determinants in the evolution of AR function from SR function. Interestingly, a smaller subset of modifications here, specifically the P153S and N154S mutations, seem to confer a much greater L-Ser racemization bias into hSR, over competing L-Ser β-elimination activity, than is seen in the native enzyme. This observation suggests that there may be an advantage to maintaining β-eliminase activity in the native enzyme, perhaps as an additional control point for L-Ser homeostasis.

As has been discussed herein, the core of the hSR enzyme features an archetypical PLP binding site; this includes the active site lysine (K56) covalently engaging the cofactor, the tetraglycine loop binding the phosphate moiety (Figure 5), and both an H-bond donor (S313) in the ring plane engaging the pyridine nitrogen and an edge-to-face π-π interacting partner residue (F55) orthogonal to the ring plane (Figure 6). In the case of the SpSR enzyme, at least, that core can apparently be modified via an unusual β-elimination-K56/conjugate addition sequence, leading to an apparent lysino-D-alanyl extender arm version of the active site (Figure 9). Surrounding this PLP binding pocket are key catalytic residues, including K56, which doubles as the *si*-face base; S84, which serves as the *re*-face base for the racemization; and R135, which appears to help position negatively charged substrates, such L-SOS and L-THA, for elimination (Figure 8), and which can be exploited for inhibitor binding, as is seen in the binding motif for malonate. The acidity of S84 may be modulated by K114 via an H-bonding network through a bound water molecule (Nelson et al., 2017).

Activity of the hSR enzyme is stimulated by both divalent cations and ATP, with the latter likely operating via an allosteric mechanism associated with a conformational change upon nucleotide binding that depends upon interactions with Q89 (Figure 4). Catalytic activity of the enzyme follows a bifurcated pathway from L-Ser to either D-Ser (racemization) or pyruvate (β-elimination), likely through a common “carbanionic intermediate,” the molecular nature and charge distribution of which is yet to be established (Figure 2). Whereas, wt-hSR favors the β-elimination reaction over the racemization reaction, this preference can be inverted through specific mutations in the hSR152-155 loop region (“triple serine loop” in AR; Table 2 and Figure 10). Elucidation of the molecular basis of this reaction pathway tuning will likely require more precise structural biological studies of appropriate (mutant) enzyme-substrate combinations in the future.

**Figure 11:**
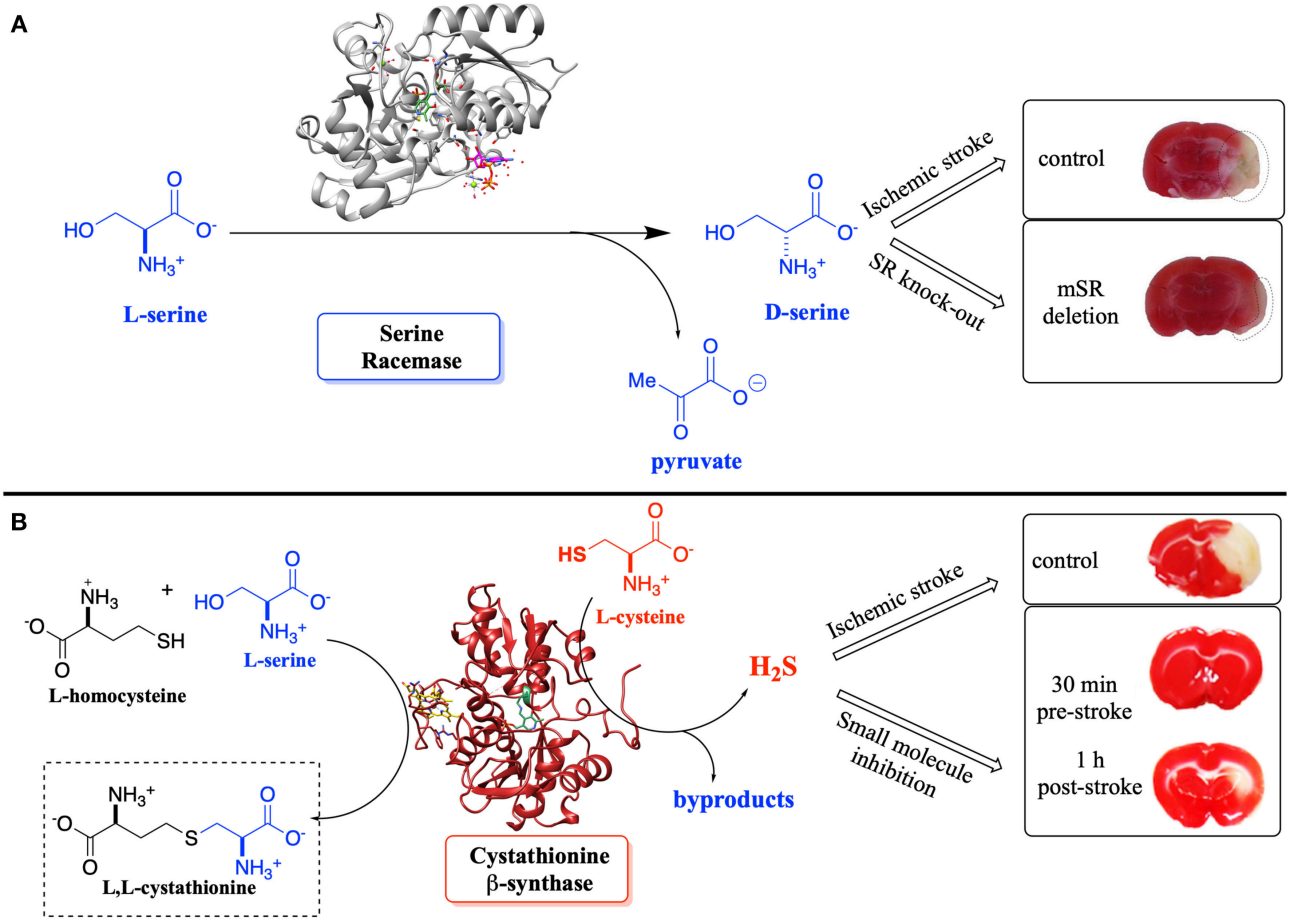
**(A)** Effect of D-Ser upon infarction volume post-ischemic stroke induced by transient middle cerebral arterial occlusion (tMCAO)–SR-knockout mice vs. control. **(B)** Effect of H_2_S upon infarction volume–inhibition of CBS vs. control; pre- and post-tMCAO. Adapted from the original articles by permission of the Society for Neuroscience (Mustafa et al., 2010) (image in **A**) and the American Chemical Society (Toney, 2005; McCune et al., 2016) (image in **B**; https://pubs.acs.org/doi/abs/10.1021/acscentsci.6b00019), respectively.

Such detailed studies of hSR structure/function relationships are critical given the importance of the enzyme in neuronal signaling via the NMDAR, in neuronal infarction pathways, and potentially in the etiology of neurodegenerative disease. Note that D-Ser and H_2_S, a product of CBS, another PLP-enzyme that controls neuronal signaling, are thought to stimulate the NMDAR (Figure 1) (Kimura, 2000, 2015; Kimura et al., 2013). Both D-Ser and H_2_S are thought to be elevated pursuant to ischemic stroke, and model studies in a tMCAO (transient middle cerebral arterial occlusion) rat stroke model suggest that both hSR (Mustafa et al., 2010) and hCBS (McCune et al., 2016) may be potential targets for inhibition to mitigate against neuronal infarction in ischemic stroke (Figure 11).

In a recent cell biology-based study on apoptosis, HEK 293T cells expressing the Q155D-hSR mutant (favoring L-Ser-to-D-Ser racemization over β-elimination) demonstrated a reduced rate of cell death when apoptotic agent staurosporine was introduced, indicating that the racemization reaction (i.e., D-Ser) may have a protective role against apoptosis (Talukdar et al., 2017). While these results are compelling, they also call out as a challenge to chemists the need to develop selective small molecule modulators of hSR that either inhibit or stimulate the enzyme or that modulate the inherent β-eliminase to racemase preference of the enzyme. This serves as motivation in our own laboratory to develop reaction-specific PLP enzyme inhibitors based upon mechanistic understanding (Berkowitz et al., 1994, 1996, 2004, 2008; Berkowitz and Smith, 1996; Karukurichi et al., 2007; McCune et al., 2016, 2017; Tu et al., 2018). If such hSR inhibitors/modulators can be developed, they will serve as tools for chemical biology, and potentially as leads for medicinal chemistry in the effort to understand hSR function in the context of neuronal signaling and D-serine neurobiology.

## Dedication

We wish to dedicate this article to Christopher T. Walsh on the 40th anniversary of his seminal treatise on enzymatic reaction mechanisms.

## Data Availability

Publicly available datasets were analyzed in this study. This data can be found here: https://www.rcsb.org/pdb/home/sitemap.do.

## Author Contributions

All authors listed have made a substantial, direct and intellectual contribution to the work, and approved it for publication.

### Conflict of Interest Statement

The authors declare that the research was conducted in the absence of any commercial or financial relationships that could be construed as a potential conflict of interest.
